# Psychometric evaluation of the Vietnamese version of nurses’ ethical behaviors for protecting patient rights scale (V-NEBPPRS): a methodological study

**DOI:** 10.1186/s12912-024-02060-2

**Published:** 2024-06-17

**Authors:** Ninh Do Thi, Gunjeong Lee, Dian Susmarini

**Affiliations:** 1 College of Nursing, Ewha Womans University, Seoul, South Korea; 2 Faculty of Nursing, Hai Phong University of Medicine and Pharmacy, Hai Phong, Vietnam; 3 Faculty of Health Sciences, Jenderal Soedirman University, Purwokerto, Indonesia

**Keywords:** Ethics, Patient rights, Nurses, Validity, Reliability, Vietnam

## Abstract

**Background:**

Recognizing patients’ rights as fundamental human rights, the global healthcare community, including the World Health Organization and various nursing organizations, has emphasized the critical role of nurses in upholding these rights through ethical practice and patient-centered care. However, in the complex landscape of healthcare, nurses in Vietnam face various ethical issues and challenges that may impede their ability to protect patient rights effectively, necessitating tools for better ethical decision-making and practice.

**Purpose:**

This study aims to translate the Nurses’ Ethical Behaviours for Protecting Patient Rights Scale (NEBPPR) into Vietnamese and evaluate the validity and reliability of the V-NEBPPRS.

**Methods:**

The original scale underwent a cross-cultural translation process to be adapted into Vietnamese. Construct validity was assessed using confirmatory factor analysis (CFA). The convergent validity, discriminant validity, and reliability of the V-NEBPPRS were evaluated.

**Results:**

After removing four items with factor loading below 0.5, the V-NEBPPRS comprises 24 items divided into five factors. CFA demonstrated a good model fit (χ2/df = 2.86; GFI = 0.87; IFI = 0.85; CFI = 0.84; RMSEA = 0.07). Convergent and discriminant validity were confirmed with extracted mean variance ranging from 0.54 to 0.67, 0.54 to 0.67, and composite reliability from 0.73 to 0.81. Cronbach’s α coefficient was 0.85 for the total scale and ranged from 0.70 to 0.79 for five subscales.

**Conclusion:**

The V-NEBPPRS is a reliable tool, providing nursing leaders and researchers with the means to utilize the V-NEBPPRS for assessing and promoting nurses’ awareness and behaviour in safeguarding patients’ rights, thereby contributing to improved overall health outcomes.

## Introduction

Patient rights are considered fundamental human rights that aim to protect patients’ dignity, integrity, and overall well-being [1]. The rights ensure that patients are treated with respect, safety, fairness, and equality throughout their healthcare experience, irrespective of their socioeconomic status, religious beliefs, gender, or ethnic background [2]. The International Council of Nurses (ICN) and nursing organizations worldwide have acknowledged the pivotal role of nurses in safeguarding patients, which is reflected in their ethical codes [3, 4].

Nurses uphold patients’ values, well-being, and autonomy and enhance their safety and overall quality of life by providing patient-centered care services and advocating for the patients based on ethical nursing principles [5–7]. Ethical principles shape and guide the ethical behavior of nurses [8]. By adhering to ethical principles, nurses can ensure that their actions are morally and ethically sound and align with the highest standards of ethical conduct [9]. Therefore, nurses must be aware of ethical principles to have the ability to apply moral reasoning in nurse care and practice ethical behaviours to protect the patient’s rights.

Nurses encounter numerous ethical issues daily in the complex healthcare context, with challenging ethical choices or unsatisfactory alternatives that may threaten patients’ rights [10, 11]. Various barriers hinder nurses from consistently exhibiting ethical behaviours in their professional practice, such as stressful work environments, time constraints, limited involvement in ethical decision-making, conflicting values or standards, and a desire to meet external expectations [12–14]. Nurses work under strict time constraints and handle a demanding workload, contributing to burnout and ethical insensitivity that may result in nurses feeling powerless and unable to deliver comprehensive care. Additionally, despite working with resource limitations, inadequate information about diseases, and organizational constraints, nurses are expected to navigate these challenges and make ethical decisions to provide high-quality nursing care [11, 15]. In certain instances, nurses were compelled to act in a manner that contradicted their perception of proper and compassionate care [16, 17]. Hence, nurses must recognize these ethical challenges within intricate clinical settings, demonstrate sound judgment, make ethically informed decisions, and behave appropriately to protect patients rights.

Furthermore, increasing advances in science and technology are providing more opportunities for patients to become aware of their rights by enhancing their information-seeking behavior through mass media and interactions with medical staff [18, 19]. Adverse medical events are one of the main problems in healthcare delivery [20] that attracts social attention and may impact patients’ privacy, beneficence, and overall rights. Therefore, nursing staff are required not only to meet the heightened demands of patients’ information-seeking but also to protect and advocate for both patients and themselves against societal judgment and accusations when unfavorable situations arise. Thus, nurses must promote awareness and ethical conduct in safeguarding patient rights and make decisions based on ethical principles, respect for autonomy, beneficence, non-maleficence, and justice [18].

In Vietnam, the regulation of nursing professional ethics standards was introduced to educate nurses about adhering to ethical norms aligned with the societal expectations of the nursing profession and assisting nurses in making ethical decisions when faced with ethical issues at clinical context [19]. These ethical standards generally cover eight dimensions in nursing practice that nurses may generally encounter during healthcare delivery, including ensuring patient safety, respecting patients and their family members, being friendly with patients and their family members, being honest at work, maintaining and enhancing professional capacity, promoting the profession’s ethical standards, being candid and united with colleagues and commit oneself to community and society [19]. In parallel with ethical standards, a self-evaluation tool has been issued to assess the level of ethical practice among staff nurses across various aspects in a clinical context. This tool is appropriate for a general evaluation of nurses’ ethical practices but does not comprehensively measure nurses’ behaviour in protecting patient rights. Therefore, a specific tool focused solely on the measurement of nurses’ behaviour in safeguarding patient rights is necessary for the Vietnamese healthcare system.

Our literature review indicated that the English version of Nurses’ Ethical Behaviours for Protecting Patient Rights Scale, developed by Turkish scholar Eyuboglu in 2020, is specifically designed to address the protection of patient rights [21]. This instrument consists of 28 items spanning five key dimensions: respecting patients’ rights to information and autonomy in decision-making, ensuring equitable care, providing beneficence and non-maleficence, honoring patient preferences and ethical values, and maintaining confidentiality and privacy. The scale’s validity and reliability have been confirmed, as evidenced by a KMO coefficient of 0.80, a Bartlett’s test result of *P* < 0.001, and a Cronbach’s alpha exceeding 0.80 [21]. Additionally, it was translated and adapted for Indonesian context, demonstrating good validity and reliability [22]. As the result, this methodological study aims to translate the Nurses’ Ethical Behaviours for Protecting Patient Rights Scale into Vietnamese and examine the validity and reliability of the V-NEBPPRS.

## Methods

### Research design

**Fig. 1:**
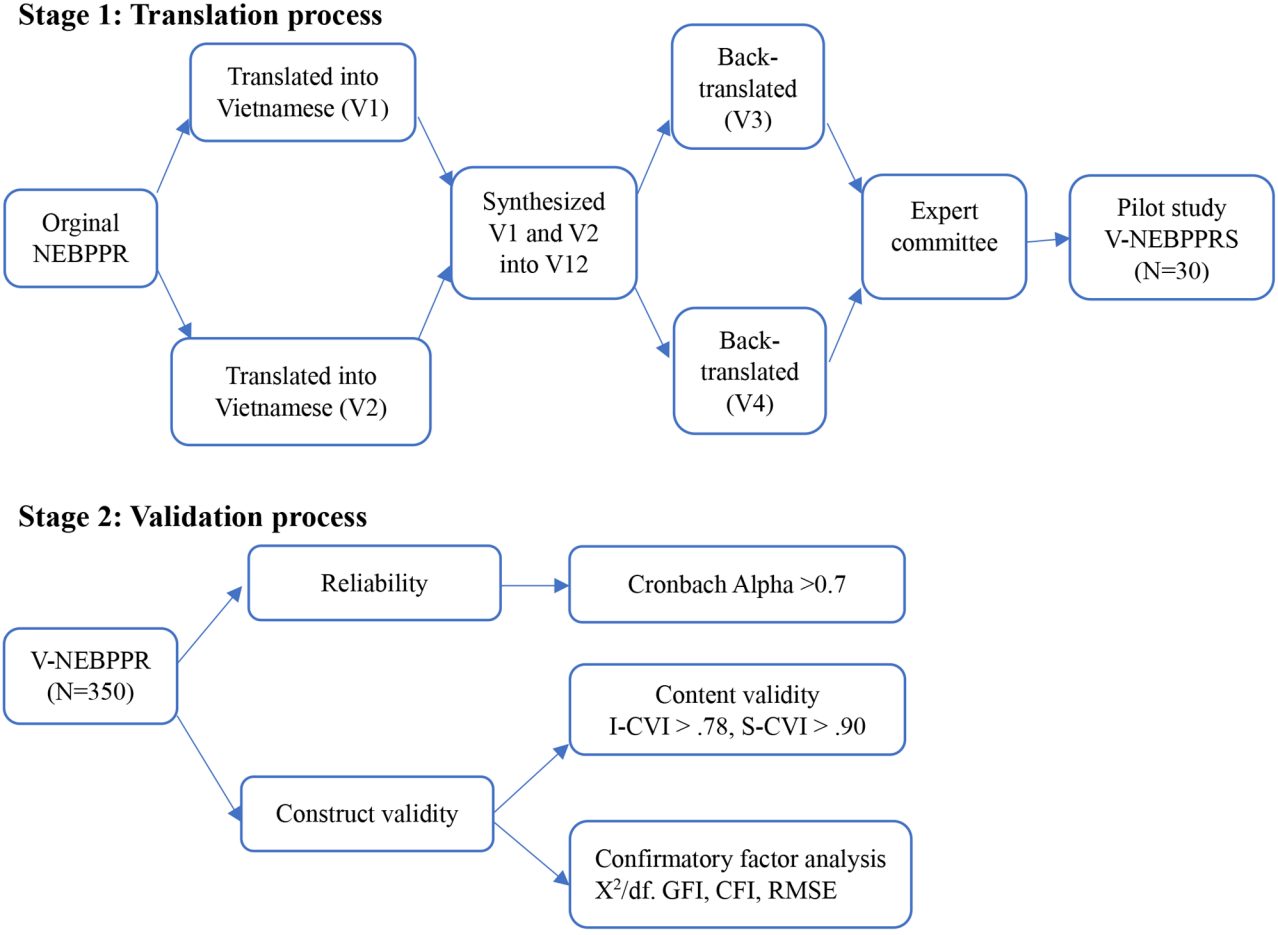
The two stages of research process

The methodological design was employed to verify the validity and reliability of the V-NEBPPRS throughout two stages [23]: the translation process of the NEBPPR into Vietnamese [24] and an examination of the validity and reliability of V-NEBPPRS. The STROBE guideline was used to report this study (Fig. 1).

### Translation process

After obtaining the author’s permission, the original BEBPPR was translated into Vietnamese using a four-step translation process [24]. Firstly, forward translation was independently conducted by two bilingual nursing professors, resulting in translated versions V1 and V2. Secondly, these two nursing professors reviewed and synthesized the translated versions to create the initial Vietnamese version, V12. Thirdly, while blinded to the original version, two other nursing lecturers independently translated V12 back into English, producing back-translation versions V3 and V4. Fourthly, an expert committee composed of researchers, language professors, and translators discussed and consolidated versions V3 and V4 to create the final Vietnamese version. They carefully evaluated each item to ensure semantic, idiomatic, and conceptual equivalence with the original version. Finally, a random sample of 30 registered nurses participated in a pilot test to confirm that the translated scale retained its equivalence in practical application [24]. No modifications were made to the scale after the pilot test.

### Validation process

#### Setting and participants

The study was conducted in two general hospitals in Vietnam between March and August 2023. The participants included nursing staff who met specific criteria: (1) voluntary participation in the study, (2) having at least one year of working experience, and (3) direct provision of patient care in the working environment. Exclusion criteria included (1) absence from the hospital due to illness or maternity leave and (2) failure to complete the questionnaire.

The appropriate sample size for confirmatory factor analysis is 5–10 times the number of scale items [25]. The scale consists of 28 items. Accordingly, this study’s estimated number of participants ranges from 140 to 280. However, to account for potential issues such as the return rate and incomplete responses, which were anticipated to be around a 15% dropout rate, the online self-administered questionnaire was distributed to 330 staff nurses across two hospitals. Out of these, 243 questionnaires were returned. Finally, 237 questionnaires were included in the data analysis after excluding six incomplete responses.

#### Instruments

The NEBPPR scale, initially developed by Eyüboglu et al. in 2022, was designed to assess nurses’ conduct in safeguarding patients’ rights. This scale comprises twenty-eight items categorized into five dimensions: (1) respect for the right to information and decision-making, (2) provision of equitable care, (3) delivering benefits without causing harm, (4) honouring patient values and preferences, and (5) safeguarding patient privacy. Respondents rated each item on a scale ranging from “never” (1) to “always” (5). The cumulative scores for all 28 items ranged from 28 to 140, with higher scores indicating more favourable ethical behaviour exhibited by nurses in protecting patients’ rights. The Cronbach’s alpha coefficient for the overall scale was 0.84, while the coefficients for the individual subscales were as follows: 0.81, 0.72, 0.67, 0.59, and 0.63.

#### Data analysis

The data was analyzed using SPSS 26.0 and AMOS 20.0 software packages. Described statistics were utilized to present the socio-demographic characteristics of the participants. Mean and standard deviation were computed for measurement data, while percentages were employed for counting data. The V-NEBPPRS’s content validity was assessed through scale-level and item-level content validity index (CVI). Internal consistency was evaluated by calculating Cronbach’s Alpha coefficient. Confirmatory Factor Analysis (CFA) was performed to assess the scale’s construct validity utilizing various fit indices to assess the goodness of fit for the dimensions. These indices included the chi-square/degrees of freedom ratio (χ²/df), the goodness of fit index (GFI), incremental fit index (IFI), Tucker-Lewis index (TLI), comparative fit index (CFI), and root mean square error of approximation (RMSEA). An acceptable model fit is indicated by χ²/df values less than or equal to 5 and RMSEA values in the range of 0.05 to 0.08. For GFI, AGFI, IFI, TLI, and CFI, values greater than 0.9 suggest an excellent model fit, while values between 0.7 and 0.9 indicate an acceptable model fit [26, 27].

To evaluate the questionnaire’s convergent and discriminant validity, standardized factor loadings, composite reliability (CR), and average variance extracted (AVE) were assessed. The standardized factor loadings exceeded 0.5, the CR values fell within the range of 0.70 to 0.95, and the AVE values were equal to or greater than 0.50 to confirm the questionnaire’s validity [28]. The Cronbach’s α coefficient was greater than 0.7, considered satisfactory for the internal consistency reliability of V-NEBPPRS [29].

## Results

### Characteristics of nursing staff

Table 1 provides an overview of the participants’ characteristics in this study. The nursing staff had an average age of 30.9 ± 5.1 years, with a mean experience of 7.9 ± 4.8 years. Among the participants, 83.5% were female, 72.2% were married, 53.6% held a diploma degree, and 42.6% possessed a bachelor’s degree.

**Table 1:** Characteristics of nursing staff (*N* = 237)

	*N* (%)	Mean (SD)
Education		
Master	1 (0.4)	
Bachelor	101 (42.6)	
Diploma	127 (53.6)	
Others	8 (3.4)	
Gender		
Male	39 (16.5)	
Female	198 (83.5)	
Marital status		
Married	171 (72.2)	
Single	58 (24.5)	
Divorced	4 (1.7)	
Others	4 (1.7)	
Working unit		
ICU	5 (2.1)	
Internal	55 (23.2)	
Pediatric	20 (8.4)	
Radiology	11 (4.6)	
Reabilitation	13 (5.5)	
Surgical	66 (27.8)	
Obstetrics	29 (12.2)	
Patient services	6 (2.5)	
Dentistry	19 (8.0)	
Otolaryngology	13 (5.5)	
Age < 30 ≥ 30	103 (43.5) 134 (56.5)	30.9 (5.1)
Clinical experience (years) < 3 3 ≤ to < 10 10 ≤	38 (16.0) 112 (47.3) 87 (36.7)	7.9 (4.8)

### Content validity

The assessment of content validity involved the evaluation of the instrument by a panel of ten nursing experts, who used a 4-point Likert scale (4 = highly relevant, 3 = moderately relevant, 2 = slightly relevant, 1 = not relevant) to rate each item. The expert panel determined the item content validity index (I-CVI) by calculating the proportion of items rated 3 or 4. The scale-level content validity index (S-CVI) was computed as the average of all the items’ content validity indexes. To establish acceptable content validity, it was ensured that S-CVI was ≥ 0.90 and I-CVI was ≥ 0.78 [29]. The findings revealed that each item had an I-CVI value ranging from 0.80 to 1.00, while the S-CVI was 0.98, indicating strong content validity for the scale. Consequently, all items were retained in the questionnaire.

### Construct validity

**Fig. 2:**
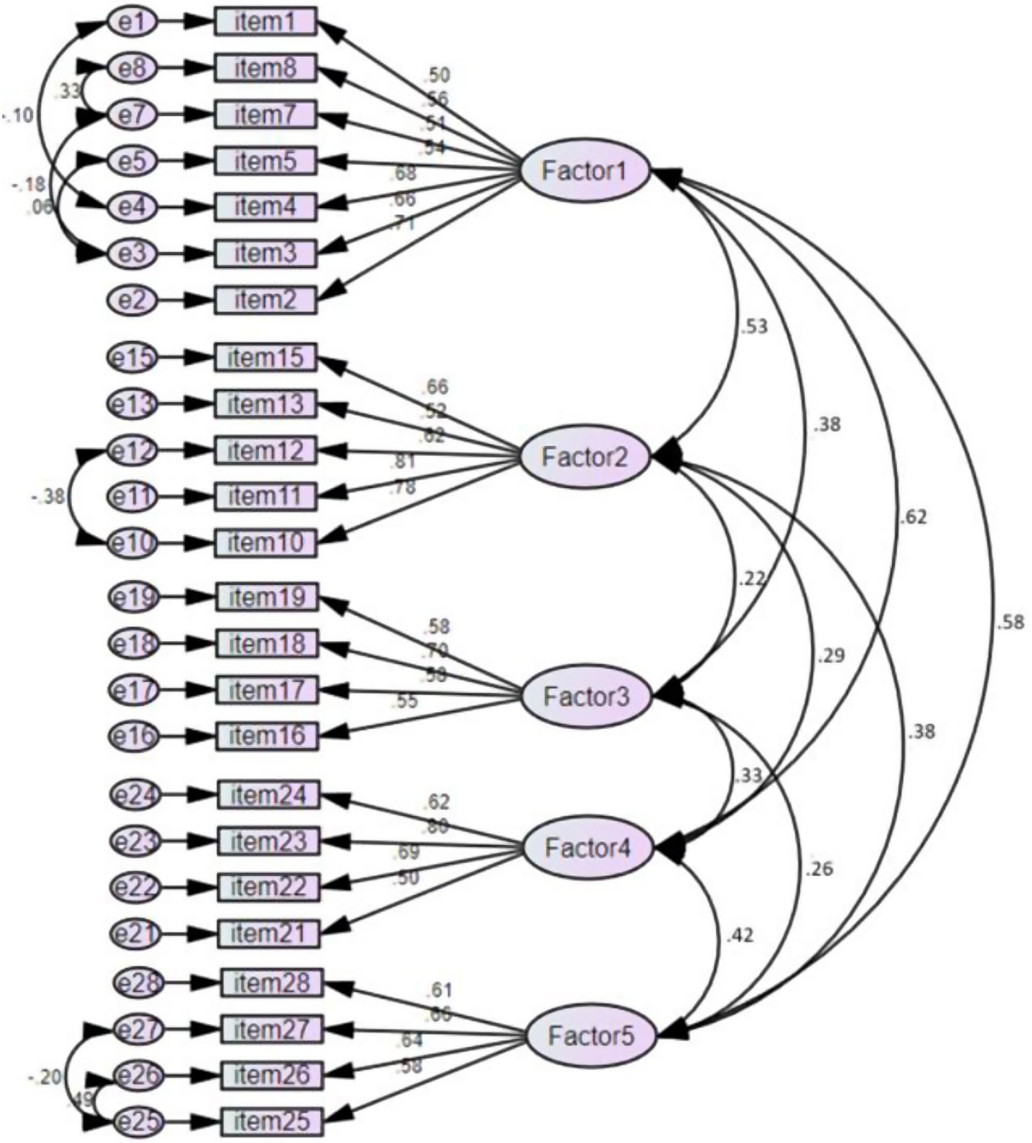
Confirmatory factor analysis of the Vietnamese version of Nurses’ Ethical Behaviors for Protecting Patient Rights Scale

The result of CFA to assess the structural relationships among five factors of the V-NEPPR is displayed in Table 2. The results indicated that the chi-square/degrees of freedom ratio (χ²/df) equals 3.86, which is less than 5. However, GFI = 0.75, IFI = 0.73, TLI = 0.79, and CFI = 0.74 values did not meet the recommended criteria of model fit (Model I). Hence, four items (item 6, item 9, item 14, item 20) with standardized factor loading coefficients below 0.5 were removed to enhance the model fit (Model II) (Table 3). Additionally, the modification index (MI > 4) [30] was employed to establish correlations between the error covariances of two items, further improving the model’s goodness of fit (Fig. 2). The results of Model II, which included 24 items, remaining original five factors, demonstrated valid values that met the requirements for model fitting, as follows: χ2/df = 2.86; GFI = 0.87; IFI = 0.85; CFI = 0.84; RMSEA = 0.07.

**Table 2:** Model goodness of fit indices of V—NEBPPRS (*N* = 237)

Models	χ2 /df	GFI	IFI	TLI	CFI	RMSEA
Model I (28 Items)	3.86	0.75	0.73	0.79	0.74	0.08
Model II (24 Items)	2.86	0.87	0.85	0.82	0.85	0.07

**Table 3:** The deleted items

Items	Factor	Contents	Factor loading
Item 6	Factor 1	I think it is not necessary to explain the practices I will perform to the patients who lost their ability to make decisions (unconscious).	-0.65
Item 9	Factor 1	I create an opportunity for the patient to take part in care and treatment decisions.	0.28
Item 14	Factor 2	I am curious about the private lives of patients.	0.36
Item 20	Factor 3	I refrain from interfering in a patient’s private life without medical reason	0.41

### Convergent and discriminant validity

Convergent validity is typically confirmed when composite reliability (CR) values equal or exceed 0.7, standardized factor loadings equal 0.5 or higher, and average variance extracted (AVE) values equal 0.5 or higher. This study’s results indicated CR values ranging between 0.73 and 0.81, standardized factor loadings values within the range of 0.5 to 0.8, and AVE values spanning from 0.54 to 0.67, thereby supporting convergent validity. Regarding discriminant validity, none of the AVE values were found to be significantly lower than the square of the correlation coefficient between the two subfactors; thus, the discriminant validity was established (Table 4).

**Table 4:** Result of CFA, convergent, discriminant validity of V—NEBPPR (*N* = 237)

Items	Factors	SE	C.*R*	*p*	Standardized Estimates (β)	AVE	CR	Factor 1 (*r*)	Factor 2 (*r*)	Factor 3 (*r*)	Factor 4 (*r*)	Factor 5 (*r*)
I1	⇓ Factor 1				0.500	0.56	0.79					
I8		0.108	7.090	< 0.001	0.561							
I7		0.110	6.592	< 0.001	0.510							
I5		0.149	6.879	< 0.001	0.544							
I4		0.131	7.639	< 0.001	0.677							
I3		0.154	7.637	< 0.001	0.663							
I2		0.138	8.002	< 0.001	0.713							
I15	⇓ Factor 2				0.657	0.58	0.78	0.530				
I13		0.103	8.617	< 0.001	0.522							
I12		0.076	9.422	< 0.001	0.619							
I11		0.124	12.362	< 0.001	0.814							
I10		0.117	11.421	< 0.001	0.777							
I19	⇓ Factor 3				0.579	0.67	0.81	0.382	0.221			
I18		0.063	9.126	< 0.001	0.698							
I17		0.075	8.132	< 0.001	0.580							
I16		0.102	7.791	< 0.001	0.545							
I24	⇓ Factor 4				0.619	0.54	0.75	0.622	0.290	0.328		
I23		0.095	10.604	< 0.001	0.795							
I22		0.082	9.853	< 0.001	0.691							
I21		0.082	7.767	< 0.001	0.505							
I28	⇓ Factor 5				0.612	0.66	0.73	0.580	0.379	0.258	0.423	
I27		0.169	8.925	< 0.001	0.657							
I26		0.151	8.780	< 0.001	0.639							
I25		0.145	7.891	< 0.001	0.583							

### Reliability

The Cronbach’s alpha reliability coefficient of V-NEBPPRS was 0.85, and factor 1, factor 2, factor 3, factor 4, and factor 5 were 0.78, 0.79, 0.75, 0.73, and 0.74, respectively (Table 5). Based on these results, the V-NEBPPRS demonstrated satisfactory internal consistency.

**Table 5:** Internal reliability of final version of V-NEBPPRS (24 items)

Factors	Items	Alpha coefficient if item deleted	Alpha coefficient
**1. Respect of the right to information and decision making (factor 1)**			0.78
I make the care-related decision with the patient	1	0.845	
I inform the patient before my professional practices	2	0.845	
I inform the patients about their rights	3	0.847	
I respect the patient’s right to know the caregiver and health professional that will provide treatment	4	0.845	
I introduce myself to the patient	5	0.844	
I receive the patient’s consent before performing my professional practices	6	0.841	
I inform the patient and/or family about the professional practices I will perform for the patient	7	0.843	
**2. Providing fair care (factor 2)**			**0.79**
I provide more attentive care for the patients whose socioeconomic levels are higher	8	0.849	
I provide more attentive care for the patients whose beliefs are similar/close to mine	9	0.845	
I refrain from providing care for the patients whose political opinions are different than mine	10	0.846	
I give priority to the families of health professionals in my professional practices	11	0.848	
I provide more attentive care for the patients whose values are similar/close to mine	12	0.847	
**3. Providing benefit-not harming (factor 3 )**			**0.75**
I assess my professional practices in terms of the risk of harming the patients	13	0.840	
I focus on providing benefit to the patient in my professional practices	14	0.843	
I take precautions against situations that may harm the patient	15	0.840	
I refrain from professional practices that have the risk of providing more harm than benefit to the patient	16	0.842	
**4. Respect for patient values and choices (factor 4)**			**0.73**
I respect a patient’s right to select the caregiver and health professional that will provide care and treatment	17	0.845	
I perform my professional practices in the framework of respect for the patient’s beliefs	18	0.841	
I respect a patient’s right to perform his/her prayers	19	0.840	
I refrain from performing professional practices refused by the patient	20	0.842	
**5. Attention to privacy (factor 5)**			**0.74**
I refrain from sharing information related to a patient’s private life with others without medical reason	21	0.839	
I refrain from sharing patient information with the people who are not involved in the care and treatment process	22	0.838	
I feel uncomfortable when the patient files are in a public place/open to all	23	0.841	
I receive the patient’s consent to get a practice done/watched on the patient with training purposes	24	0.838	
**Total**			**0.85**

## Discussion

The NEBPPR was translated into Vietnamese, and exhibited validity and reliability for evaluating the ethical behavior of nursing staff in Vietnam regarding the protection of patients’ rights. The Vietnamese version of NEBPPR consists of 24 items divided into five dimensions: respect for the right to information and decision-making, providing fair care, providing benefits not harming, respect for patient values and choices, and attention to privacy. This instrument holds the potential to facilitate future research to enhance nurses’ awareness of patients’ rights, thereby promoting ethical behaviour in safeguarding those rights. A valid and reliable measurement scale can offer insights into how well nurses who have been trained in specific patients’ rights and the corresponding protective behaviours have assimilated this knowledge. This information can be instrumental in designing initiatives to enhance nurses’ ethical practices in safeguarding patients’ rights [21].

The results for content validity indicated that both the S-CVI and I-CVI indexes met the criteria [31], without changes recommended by the committee. Therefore, the instrument was appropriately translated for the target population.

The CFA conducted in this study demonstrated that the V-NEBPPRS achieved structural validity within the Vietnamese nursing context. The tool’s five-factor structure was not only verified but also found to be consistent with the original NEBPPR’s five-factor structure [21]. This consistency extends to the recently translated Indonesian version (I-NEBPPR) [22] and Korean version [32]. The scale effectively reflects the adequate dimensions of the measured construct, thereby providing substantial support for the structural validity of the V-NEBPPRS.

There were differences in the number of items among the original 28-item Turkish, 23-item Indonesian, and 24-item Vietnamese versions. This variation may be attributed to exploratory factor analysis (EFA), used in the original Turkish version, while CFA was employed in the three translated versions to support the instrument’s structure and items. The EFA is typically used in the initial stages to explore the possible underlying factor structure of a dataset without imposing a preconceived structure [26], which aligns with the development stage of the original instrument in Turkey. CFA was subsequently employed in the translated versions to rigorously test the stability and applicability of the factor structure identified through EFA in the Turkish version in new cultural contexts. This approach helps to ensure the validity and reliability of the instrument across different languages and settings [26]. Additionally, cultural differences could have affected the unequal number of items among the three versions [33]. For instance, a distinct cultural concept called ‘Siri na passe,’ prevalent among the Bugis population in Indonesia, significantly influences daily behaviors including diligence, integrity, teamwork, and conscientiousness. Bugis descent nurses often rigorously follow this ethical paradigm [22]. In Vietnam, the concept of ‘hiếu’—a fundamental element of Confucianism—emphasizes filial piety and ethical behaviors dealing with family members, community and society [34]. This principle plays a significant role in shaping ethical behaviors and is deeply ingrained in Vietnamese culture, influencing professional conduct across various fields, including healthcare. Guided by ‘hiếu,’ Vietnamese nurses show heightened respect towards patients and demonstrate a meticulous approach to patient care.

The V-NEBPPRS eliminated four items (items 6, 9, 14, and 20) with standardized factor loadings below 0.5. Item 6, which expressed that nurses do not need to explain the care plan to patients who have lost their decision-making ability, conflicted with the practice of nurses providing relevant information to patients before implementing nursing interventions, even if the patients are unconscious. This practice is in line with the ethical standards of the Vietnamese nursing profession [35] and is commonly observed among Vietnamese nurses. Item 9, ‘I create an opportunity for the patient to take part in care and treatment decisions,’ was removed because it focuses on the nurse’s role in facilitating patient autonomy. This role primarily involves ensuring that patients have the opportunities and necessary information to make informed decisions themselves. It reflects the respect nurses have for patients’ participation in care and decision-making. Practically, the content of item 9 was already covered under the ‘respect the patient’s right to self-determination in providing care’ subdimension of the ‘respect patient and their family member’ dimension in Vietnamese ethical standards [35]. Items 14 and 20 were also removed because their standardized factor loadings were less than 0.5. Both items focus on improper curiosity about the patient’s life, a topic also addressed in the ethical standards of the Vietnamese nursing profession [35]. Vietnamese nurses are likely highly familiar with and adhere to these standards. This finding is consistent with the results reported in Susmarini’s studies in 2023, which noted that the fundamental elements covered by these items are thoroughly examined by item 15, which assesses nurses’ respect for patients with diverse values.

The final model includes 24 items and was confirmed through an appropriate model confirmation process. Although four items were removed from the original instrument, the V-NEBPPRS still aligned with the overall concept of nurses’ ethical behaviour in protecting patients’ rights. Additionally, this model’s suitability was similar to that reported in previous studies [22, 32]. Moreover, in the relationships among factors of Model II, the significance (C.R) values ranged from 6.9 to 12.3; constituent variables significantly explained all factors at a significance level of 0.05. Therefore, the V-NEBPPRS with 24 items was accepted as the final instrument.

Convergent validity pertains to the alignment of indicators measuring the same construct [36]. The findings of this study affirm the fulfilment of the criteria, with CR, standardized factor loadings and AVE values spanning from 0.73 to 0.81, 0.5 to 0.8, and 0.54 to 0.67, respectively. Consequently, the convergent validity of the V-NEBPPS was confirmed. Discriminant validity is generally described as “two distinct constructs” and is evaluated by the correlation between the two constructs [37]. The study results showed that none of the AVE values were found to be significantly lower than the square of the correlation coefficient between two subfactors. Hence, the discriminant validity of the V-NEBPP instrument was successfully established. This study’s convergent and discriminant validity results were consistent with studies of Susmarini et al. [22] and Yun [32]. However, it is impossible to compare to the original version by Egyboglu [21], which did not report convergent and discriminant validity.

In this study, the V_NEBPPRS’s Cronbach’s coefficient was 0.85, with subscales ranging between 0.73 and 0.79, indicating good internal consistency and homogeneity. Notably, these Cronbach’s α values were higher than those reported in the original study, possibly due to cultural differences.

The final version of the 24-item V-NEBPPRS included five factors (Table 5) and utilized a five-point Likert scale ranging from “never” to “always”, with scores from one to five. Factor 1, “Respect for the right to information and decision-making” consists of seven items. Factor 2, “Providing fair care” comprises five items requiring reverse coding. Factor 3, “Providing benefit - not harming” includes four items, and both Factor 4, “Respect for patient values and choices” and Factor 5, “Attention to privacy” each include four items.

### Limitations

This study also exists its limitations. The study collected data using self-administered measures, which may introduce the potential for social desirability response bias [38], especially when assessing moral behaviour. Therefore, further research employing the V-NEPPR to explore the concept, aspects, and factors influencing ethical behaviour in protecting patients’ rights through integrated observation and self-report data is essential. This approach helps mitigate methodological bias and enhances our understanding of nurses’ ethical behaviour, thereby improving healthcare service quality.

## Conclusion

The V-NEBPPRS was developed in accordance with the Vietnamese nursing context through a translation process to assess the ethical behaviour of nurses in protecting patients’ rights. The 24-item V-NEBPPRS is divided into five factors and employs a 5-point Likert scale for assessment. The study confirmed content validity, construct validity, convergent validity, discriminant validity, and reliability of the V-NEBPPRS. Therefore, the V-NEBPPRS is a reliable tool for evaluating the ethical behaviour of nurses in protecting patients’ rights in Vietnam. The instrument will provide nursing leaders and researchers with the means to utilize the V-NEBPPRS for assessing and promoting nurses’ awareness and behaviour in safeguarding patients’ rights. Additionally, nurse educators can effectively utilize the V-NEBPPRS to assess and cultivate ethical behavior and decision-making skills among nursing students. This will not only help in reinforcing the importance of safeguarding patients’ rights from the early stages of their professional education but also in promoting a culture of ethical awareness and responsibility, thereby contributing to improved overall health outcomes.

## Data Availability

Data are available upon request from the author at the following email dtninh90@gmail.com.
